# Protocol for identifying cellular and molecular signatures from spatial profiling data using micro-tissue analysis

**DOI:** 10.1016/j.xpro.2026.104825

**Published:** 2026-09-18

**Authors:** Huiqian Hu, Alphonsus H.C. Ng, Yue Lu

**Affiliations:** 1 Department of Molecular Pharmaceutics, University of Utah, Salt Lake City, UT 84112, USA; 2 Department of Biomedical Engineering, University of Utah, Salt Lake City, UT 84112, USA

**Keywords:** Biotechnology and bioengineering, Cancer, Health Sciences, Immunology, Microbiology, Microscopy, Proteomics, Sequence analysis, Sequencing, Single Cell, Systems biology

## Abstract

Spatial tissue profiling measures hundreds to thousands of biomolecules across tissue regions, but most analyses examine each feature one at a time. Here, we present a protocol for micro-tissue analysis using partial least-squares modeling. We describe steps for stratifying regions by microenvironment, building multivariate models to identify which features collectively drive a biological outcome, and interpreting the results. We detail four worked examples using two published GeoMx digital spatial profiler (DSP) datasets from human tumor and mucosal tissues.

For complete details on the use and execution of this protocol, please refer to Lu et al.^1^ and Ng et al.^2^

## Highlights

• A framework for using micro-tissue regions to find what drives a biological outcome
• Steps for stratifying regions by microenvironment and modeling many features together
• Instructions for validating predictions at the tissue and patient level
• Reproducible R workflow with four worked examples on spatial profiling data

## Abstract (editor highlights)

Publisher’s note: Undertaking any experimental protocol requires adherence to local institutional guidelines for laboratory safety and ethics.

## Graphical abstract

**Figure:**
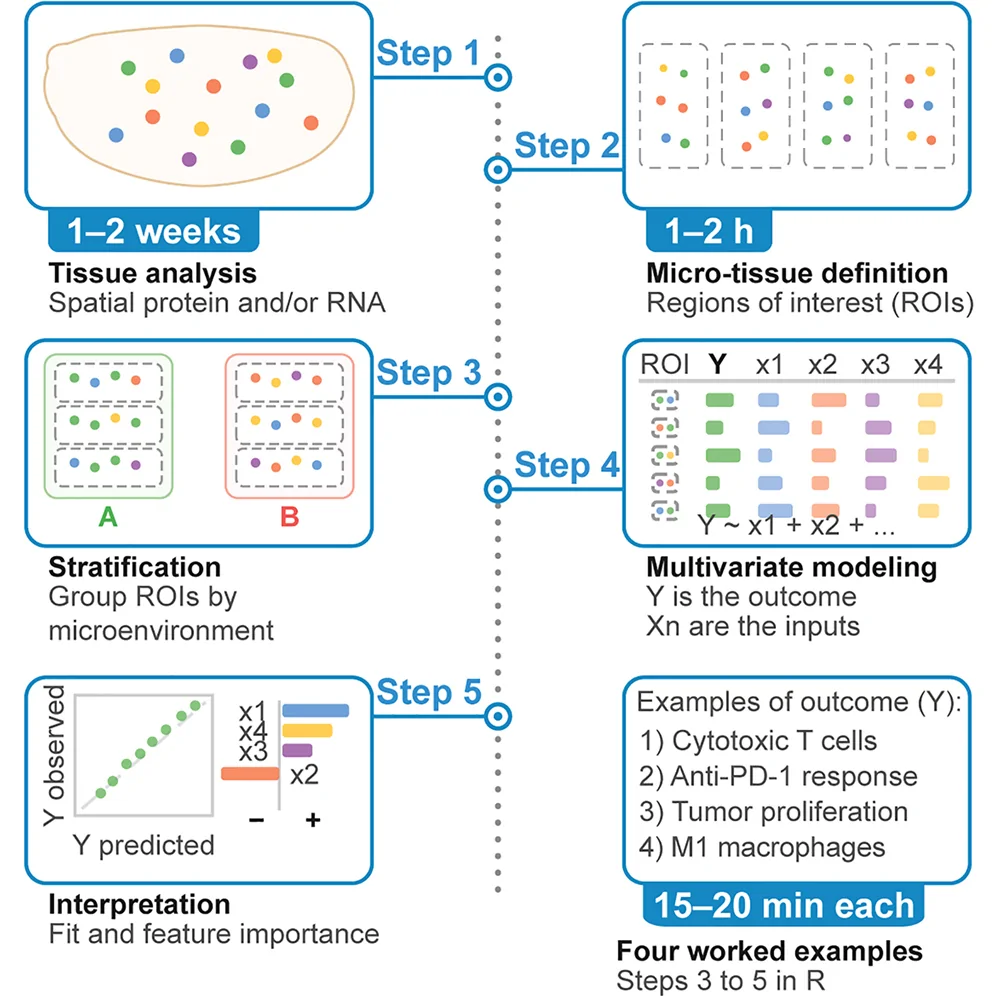

## Before you begin

**Timing: The full computational workflow takes approximately 1–2 h once data and software are prepared**

This protocol describes micro-tissue analysis, a framework that uses the biological variation between tissue regions as the basis for modeling. Rather than averaging across regions to produce a single tissue-level summary, this approach retains each region as a separate data point, stratifies regions by microenvironment, and models all features together to identify which collectively drive treatment response or other biological outcomes. The protocol begins after tissue profiling has been completed, and thus, the methods described here are purely computational.

This protocol uses partial least squares (PLS) as the multivariate modeling method.^3^ PLS finds combinations of measured features (called latent components) that best predict an outcome variable. When the outcome is continuous (e.g., protein expression or cell-type abundance), the method is PLS regression (PLS-R); when the outcome is a categorical label (e.g., responder vs. nonresponder), it is PLS discriminant analysis (PLS-DA). Each feature receives a Variable Importance in Projection (VIP) score quantifying its contribution to the model; features with VIP > 1 are conventionally considered important. Model quality is assessed by R^2^X (variance in predictors captured), R^2^Y (variance in outcome explained), and Q^2^ (variance predicted by cross-validation). These metrics and the VIP-coefficient plot are described further in quantification and statistical analysis.

We demonstrate the protocol using two published datasets generated on the NanoString (Bruker) GeoMx Digital Spatial Profiler (DSP). GeoMx DSP enables high-plex molecular profiling of user-selected regions within intact tissue sections: the tissue is stained with fluorescent markers to visualize morphology and cell populations, regions of interest (ROIs) are selected based on the fluorescence pattern, and UV-cleavable barcoded probes within each region are released and counted to quantify protein or RNA targets.^4^ The two demonstration datasets are: (1) a 35-protein immuno-oncology panel profiled across glioblastoma tumors treated with anti-PD-1 immunotherapy in the neoadjuvant setting,^1^ and (2) a 1,833-transcript cancer transcriptome panel profiled across lung and gut autopsy tissue from a COVID-19 case.^2^ In the four worked examples described in this protocol, the response Y is CD8A expression, representing cytotoxic T-cell infiltration (worked example 1); the anti-PD-1 treatment response, defined by an independent bulk molecular signature (worked example 2); Ki67 expression, representing tumor proliferation in immune-poor regions (worked example 3); and the abundance of M1 macrophages, representing inflammatory macrophage infiltration (worked example 4). The predictors X are the other measured proteins or cell types. Although demonstrated here on GeoMx DSP data, the analytical framework is applicable to region-level expression data from other spatial profiling technologies.

### Innovation

In ROI-based spatial profiling, the most common analytical approach remains testing features one at a time to identify which genes or proteins differ between groups. This reveals what changed, but not how features relate to each other or what drives the biological variation between regions within a tissue. Region-level heterogeneity is typically treated as noise to be averaged away rather than signal to be leveraged. Although multivariate methods for predicting clinical outcomes from spatial data are increasingly reported in the literature, this type of analysis remains underutilized relative to its potential.

Micro-tissue analysis retains each region of interest as a separate data point rather than averaging across regions to produce a single tissue-level summary. This preserves the biological variation between regions, which is the basis for all downstream modeling. Two analytical principles follow. First, regions should be stratified by biological context before modeling. For example, separating immune-rich from immune-poor compartments reveals that different microenvironments follow different biological rules and require separate models. Second, all key features should be evaluated simultaneously rather than one at a time. Multivariate modeling captures interrelationships among molecular and cellular features, identifying which combinations collectively distinguish an outcome such as treatment response, and in which direction.

This protocol makes the framework accessible by providing a reproducible, end-to-end workflow with open-source R code and worked examples.

### Institutional permissions

This protocol is purely computational and does not involve any new experiments on live vertebrates or human subjects. No institutional ethics approval is required to perform the analysis described here.

The two demonstration datasets were generated from human tissue under ethics approvals described in the original publications.^1^^,^^2^ Researchers who generate their own spatial profiling data from human or animal tissue should obtain the necessary ethics approvals and informed consent, as appropriate, from their institutional review board or equivalent regulatory body before tissue collection and profiling.

### ROI selection, fluorescent staining, and spatial profiling

**Timing: 1–2 weeks (tissue preparation, instrument run, and barcode readout)**

This preparation covers the wet-lab steps that must be completed before the computational analysis can begin. The GeoMx DSP workflow consists of staining tissue sections with both barcoded probes and fluorescent morphology markers, scanning and selecting ROIs on the instrument, UV-cleaving the barcodes from each selected region, and reading out the released barcodes by nCounter or next-generation sequencing. The steps below describe the key decisions at each stage. For detailed bench procedures, refer to the GeoMx DSP Manual Slide Preparation User Manual (NanoString/Bruker, MAN-10150), Merritt et al.,^4^ and Kim et al.^5^

1. Prepare formalin-fixed, paraffin-embedded (FFPE) (or frozen) tissue sections and stain with both the barcoded probe panel and fluorescent morphology markers in a single slide preparation workflow, following the protocol for your assay type.
  a. For protein assays, apply the oligonucleotide-conjugated antibody cocktail together with fluorescent morphology markers. For RNA assays (e.g., Cancer Transcriptome Atlas [CTA] or Whole Transcriptome Atlas [WTA]), hybridize the barcoded RNA probe panel to the tissue sections, then apply fluorescent morphology markers.
  b. Select up to four morphology markers for the GeoMx DSP.
    ***Note:*** Typically a nuclear stain (SYTO 13) plus up to three fluorescent antibodies. Choose markers that visualize the tissue compartments relevant to your biological question (e.g., CD45 for immune cells, PanCK for epithelial cells, GFAP for astrocytes, S100B for melanoma cells).
  c. If fluorescent antibody markers are insufficient or unavailable for your tissue type, use adjacent H&E-stained sections to guide ROI selection based on histological features such as tissue architecture, lymphoid aggregates, or pathological changes.
  d. Also consider using RNAscope probes for visualizing specific transcripts in situ (e.g., viral RNA, cell-type-specific transcripts), though signal quality may vary by target, tissue type, and specimen quality.
    ***Note:*** Fluorescent antibody morphology markers are compatible with both protein and RNA assays on the GeoMx DSP. The morphology markers are used for visualization and ROI selection only; they are independent of the barcoded probe panel that generates the expression data.
    **CRITICAL:** Both the barcoded probe panel and the morphology markers are applied during tissue preparation, before the slide is loaded onto the GeoMx instrument. ROI selection occurs after staining is complete.
2. Scan the stained tissue on the GeoMx DSP instrument and select ROIs. Design the ROI selection strategy to capture the regional variation relevant to your biological question.
  a. Choose an ROI selection strategy that matches your biological question.
    ***Note:*** One way to compare immune and non-immune compartments is to define ROIs by thresholding a fluorescent immune marker such as CD45. In Amaria et al.,^6^ custom CD45 fluorescence masks were generated using an ImageJ script applied to thresholded CD45 fluorescence micrographs, delineating immune and melanoma tumor compartments within each tissue section.
    ***Note:*** To survey regional heterogeneity across a large tissue section sparsely infiltrated by immune cells, place geometric ROIs at representative locations spanning areas of high and low immune infiltration. In Lu et al.,^1^ geometric ROIs were used for glioblastoma (GBM) sections because the tumors had fewer, smaller immune clusters compared to melanoma. ROIs with >=25% CD45-positive area by image analysis were classified as immune-rich.
    ***Note:*** To compare anatomically distinct regions (e.g., lymphoid versus non-lymphoid tissue), select ROIs based on morphological features observed in the fluorescence scan or in adjacent H&E images. In Ng et al.,^2^ ROIs were selected based on nuclei features and pathological observations in adjacent H&E-stained sections.
  b. Collect enough ROIs per experimental group to support downstream statistical modeling.
    ***Note:*** In the demonstration datasets, Lu et al.^1^ profiled 168 ROIs across 14 GBM tumors from 13 patients, and Ng et al.^2^ profiled 53 ROIs across lung (21 ROIs) and gut (32 ROIs) tissue sections from a single COVID-19 autopsy case. The number and size of ROIs depend on tissue availability, the instrument configuration, and the biological question. Consult the GeoMx DSP Experimental Design Guide (Bruker) for recommendations on ROI sizing and number.
3. Complete the spatial profiling run following the instrument protocol for your assay type.

***Note:*** On the GeoMx DSP, UV light cleaves the barcoded probes from each selected ROI, and the released barcodes are collected and quantified by nCounter or next-generation sequencing. For detailed wet-lab procedures, refer to Kim et al.^5^ and the GeoMx DSP Instrument User Manual (NanoString/Bruker, MAN-10152).

***Note:*** In the two demonstration datasets, different tissue types, sample sizes, and ROI selection strategies were used. In Lu et al.,^1^ FFPE tumor sections were collected from 13 patients with recurrent GBM who received neoadjuvant anti-PD-1 before surgical resection, yielding 18 tumor specimens (14 of which were profiled by GeoMx DSP with a 35-plex protein panel). Tissue sections were stained with SYTO 13, CD45, and GFAP, and geometric ROIs were placed across each tumor to capture representative areas of high and low immune infiltration; 168 ROIs were analyzed.

***Note:*** In Ng et al.,^2^ FFPE autopsy tissue sections from a patient who succumbed to severe COVID-19 were first screened across six organs (brain, lung, heart, liver, kidney, and gut). The two organs with the highest viral burden (lung and gut) were selected for spatial transcript profiling using a pre-commercial version of the Cancer Transcriptome Atlas (∼1,800 RNA targets). Lung and gut sections were stained with SYTO 13 (nuclei) for the GeoMx run, and 53 ROIs were selected across both organs (21 in the lung and 32 in the gut) based on nuclei features and pathological observations in adjacent H&E-stained sections.

***Note:*** The ROIs included a mixture of lymphoid aggregates and non-lymphoid tissues in each organ. Immune categorization (immune-high versus immune-low) was determined computationally using counts of *PTPRC* (Protein Tyrosine Phosphatase Receptor Type C, encoding the pan-leukocyte marker CD45) and absolute immune score from CIBERSORTx deconvolution^7^; the deconvolution output files are included in this paper.

**CRITICAL:** Spatial profiling must be completed before beginning this computational protocol. The protocol assumes you already have the normalized expression data for your tissue sections.

### Data formatting and normalization

**Timing:** 1–2 h

4. Export the normalized expression data from your spatial profiling platform.

***Note:*** For the current commercial GeoMx DSP, quality control, target filtering, normalization, and data export can be performed within the GeoMx DSP Analysis Suite software (see Kim et al.^5^ for a step-by-step walkthrough).

5. Format the data as a table where rows correspond to individual ROIs and columns correspond to genes or proteins.
  a. Include metadata columns (e.g., sample ID, ROI number, tissue type) with the expression value columns.

***Note:*** Each cell contains the normalized expression value for that ROI-biomolecule pair.

6. Verify that the expression columns contain no missing values (NA) or blank cells. Remove or impute any incomplete rows before proceeding.
7. Save the formatted table as an Excel file (.xlsx) in a working directory accessible to R.
8. Use the two demonstration datasets provided, from Lu et al.^1^ and Ng et al.^2^ (see key resources table). Both datasets are included in the analysis repository; no separate download is required.

**CRITICAL:** The expression matrix must have ROIs as rows and genes/proteins as columns. If your instrument exports the data in the transposed orientation, rearrange it before proceeding.

***Note:*** The demonstration datasets in this protocol were generated on pre-commercial versions of the GeoMx DSP instrument, and normalization and target filtering were performed manually outside the instrument software. Figure S1 gives the recommended quality-control and normalization sequence, with the criteria for each step, and the formatted matrix it produces.

***Note:*** In Lu et al.,^1^ raw protein counts were processed in three steps using Microsoft Excel: (1) normalized with ERCC spike-in controls, (2) background subtracted by IgG isotype control counts for each ROI, and (3) normalized by the UV-light mask area to yield count density. In Ng et al.,^2^ transcript data were scaled to have the same 75th percentile of expression, which effectively normalizes by ROI area; genes below the limit of detection in more than 85% of ROIs were also excluded.

***Note:*** If you are using the current commercial platform, the instrument software handles QC, target filtering, normalization, and export, and the open-source GeomxTools Bioconductor package performs the same steps. For other platforms, use the quality-control and normalization method recommended for that assay.

### Software installation and analysis setup

**Timing: 10–15 min**

9. Install R (version 4.0 or later) from CRAN (https://cran.r-project.org).
10. Install RStudio Desktop from Posit (https://posit.co/download/rstudio-desktop).
11. Open RStudio and confirm it is using the newly installed R version by running the following command in the R console:

R.version.string

12. Install the required R packages by running the following command in the R console:

install.packages(c(“here”, “readxl”, “pls”, “ggplot2”, “pROC”, “dplyr”, “ggrepel”, “patchwork”, “viridis”, “readr”, “tidyr”, “jsonlite”, “png”))

Troubleshooting 1.

13. Download the analysis repository (see key resources table for the link).
  a. On the GitHub page, click the green Code button.
  b. Click Download ZIP.
  c. Extract the ZIP file to a location of your choice (e.g., your Desktop or Documents folder).

***Note:*** The repository contains the R scripts, both demonstration datasets, and an RStudio project file. Alternatively, if you are familiar with Git, you can clone the repository from the command line.

14. Open the file Digipharma-Micro-tissue-analysis.Rproj in RStudio.

***Note:*** This sets the working directory to the repository folder automatically, so that all file paths in the scripts resolve correctly.

**CRITICAL:** Do not use setwd() manually if you are using the.Rproj file. Troubleshooting 2.

**CRITICAL:** Ensure that the R version and all packages are up to date. Package version conflicts are the most common source of errors when running the scripts for the first time.

## Key resources table

**Table:** 

REAGENT or RESOURCE	SOURCE	IDENTIFIER
**Deposited data**		

GBM spatial protein dataset	Lu et al.^1^	https://doi.org/10.1038/s41467-021-24293-4 (Supplementary information, Dataset 1)
COVID-19 spatial transcript dataset	Ng et al.^2^	Mendeley Data: https://doi.org/10.17632/hsddc6nmzw.1
Human breast cancer Visium dataset (Block A Section 1)	10x Genomics^8^	https://www.10xgenomics.com/datasets/human-breast-cancer-block-a-section-1-1-standard-1-1-0
Analysis scripts (this protocol)	This paper	Zenodo: https://doi.org/10.5281/zenodo.19967582 GitHub: https://github.com/DigiPharma/Digipharma-Micro-tissue-analysis

**Software and algorithms**		

R (version 4.0 or later)	R Core Team	https://CRAN.R-project.org/
RStudio	Posit	https://posit.co/download/rstudio-desktop
here (R package)	CRAN	https://CRAN.R-project.org/package=here
readxl (R package)	CRAN	https://CRAN.R-project.org/package=readxl
dplyr (R package)	CRAN	https://CRAN.R-project.org/package=dplyr
pls (R package)	CRAN	https://CRAN.R-project.org/package=pls
ggplot2 (R package)	CRAN	https://CRAN.R-project.org/package=ggplot2
ggrepel (R package)	CRAN	https://CRAN.R-project.org/package=ggrepel
patchwork (R package)	CRAN	https://CRAN.R-project.org/package=patchwork
pROC (R package)	CRAN	https://CRAN.R-project.org/package=pROC
viridis (R package)	CRAN	https://CRAN.R-project.org/package=viridis
readr (R package)	CRAN	https://CRAN.R-project.org/package=readr
tidyr (R package)	CRAN	https://CRAN.R-project.org/package=tidyr
jsonlite (R package)	CRAN	https://CRAN.R-project.org/package=jsonlite
png (R package)	CRAN	https://CRAN.R-project.org/package=png
Python (version 3.10 or later)	Python Software Foundation	https://www.python.org
scanpy (Python package)	PyPI	https://pypi.org/project/scanpy/
scikit-misc (Python package)	PyPI	https://pypi.org/project/scikit-misc/
pandas (Python package)	PyPI	https://pypi.org/project/pandas/
NumPy (Python package)	PyPI	https://pypi.org/project/numpy/
SciPy (Python package)	PyPI	https://pypi.org/project/scipy/
CIBERSORTx	Newman et al.^7^	https://cibersortx.stanford.edu

## Step-by-step method details

The micro-tissue analysis workflow follows five steps (see graphical abstract): spatial profiling, micro-tissue definition, microenvironment stratification, multivariate modeling, and validation. The four worked examples below each walk through microenvironment stratification, multivariate modeling, and interpretation on a different biological question using the demonstration datasets (Table 1).

***Note:*** Microenvironment stratification requires a quantitative measure of the local microenvironment for each ROI. How you obtain that measure depends on your data and platform. If you have fluorescence imaging alongside your expression data, cell-type ratios can be quantified from the images using software such as CellProfiler,^9^ QuPath, HALO, or similar tools; this image analysis is a separate undertaking not covered in this protocol. If your data are transcript-level, computational deconvolution (e.g., CIBERSORTx) can estimate cell-type abundances from the expression matrix; the fourth worked example builds a PLS-R model on pre-computed cell abundances based on deconvolution. In the GBM protein dataset used for the first three worked examples, the ratio of CD45+ cells per ROI was pre-computed from fluorescence images by Lu et al.^1^ and is already included in the downloaded data file.

**CRITICAL:** Before running the worked examples, verify that the downloaded repository contains the expected folder structure by consulting the README file included in the repository. The repository is organized into the following folders: “R/” contains the four analysis scripts (one per worked example) and the two supplemental-figure scripts used in expected outcomes and in quantification and statistical analysis, “Nat commun data/” contains the GBM spatial protein dataset from Lu et al.^1^ (three Excel files: the full expression matrix and two pre-computed intermediate files for the PLS-DA variable selection in worked example 2), “Cell Rep data/” contains the CIBERSORTx-derived immune cell-type abundances from the COVID-19 spatial transcript dataset of Ng et al.^2^ (one CSV and one Excel file), “visium_demo/” contains the Python preprocessing for the Visium demonstration in Figure S2, and “figures/” is the output folder where the scripts write the generated figure panels.

**Table 1:** Overview of the four worked examples

Example	Script	Analysis	PLS variant	Figure
1	Figure 1.R	Protein correlates of CD8A in immune-rich GBM ROIs	PLS-R	Figure 1
2	Figure 2.R	Protein signature for classifying treatment response	PLS-DA	Figure 2
3	Figure 3.R	Multivariate signature in immune-poor GBM ROIs	PLS-R	Figure 3
4	Figure 4.R	Immune-cell correlates of M1 macrophages in lung and gut	PLS-R	Figure 4

**Figure 1:**
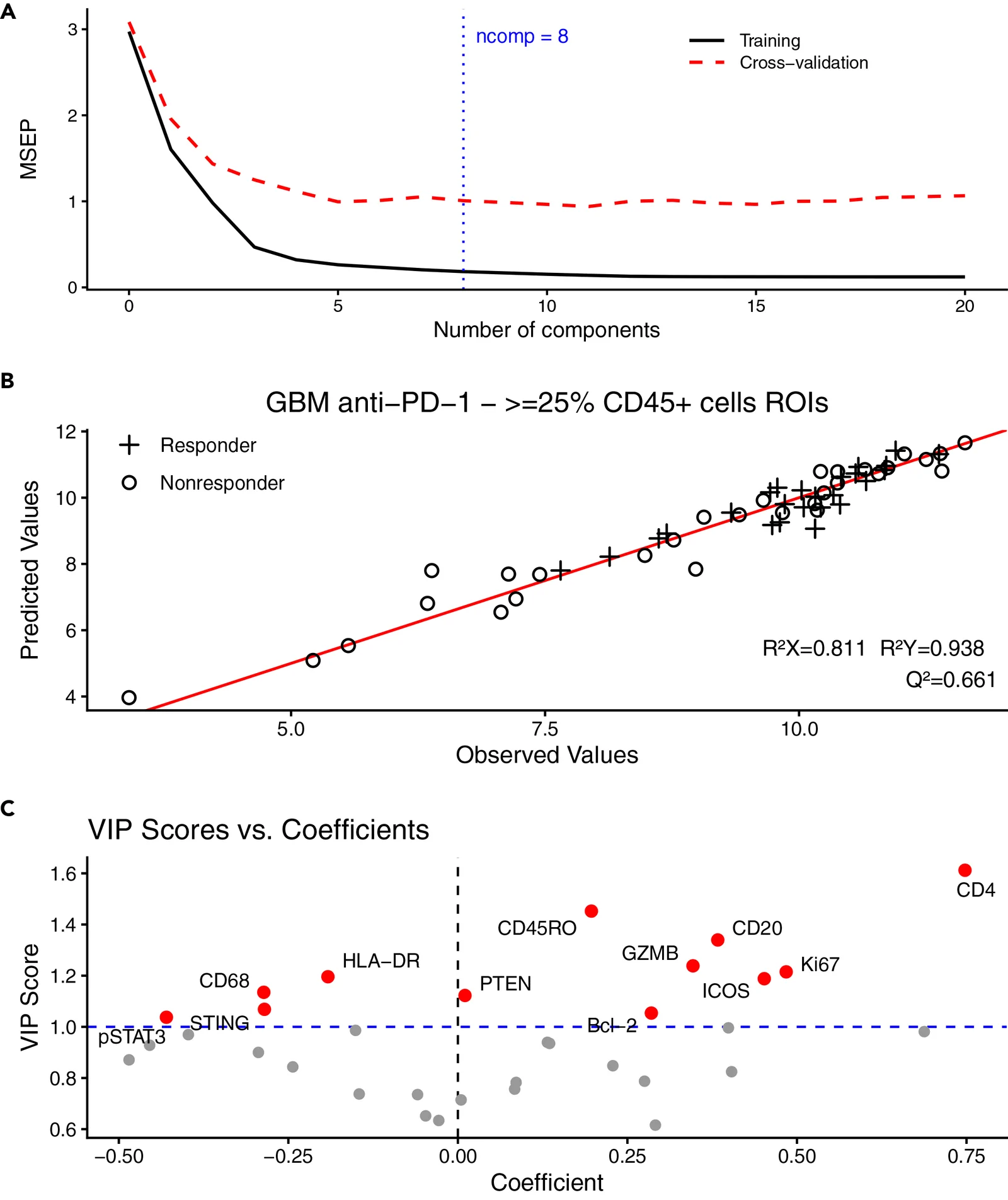
PLS-R identifies multivariate correlates of CD8 T-cells in immune-rich GBM ROIs (A) MSEP (mean squared error of prediction) curve showing training error (solid black) and 10-fold cross-validation error (dashed red) as a function of the number of PLS components. The dotted blue line indicates the selected ncomp = 8, which falls within the plateau region of the cross-validation curve. (B) Observed versus predicted CD8A expression (ln-transformed count density) for immune-rich ROIs (>=25% CD45+ cells) from GBM tumors treated with neoadjuvant anti-PD-1. Points are shape-coded by molecular response (cross = responder, circle = nonresponder). The red line is the identity line. Model-quality metrics are annotated. (C) VIP-coefficient plot. Each point represents one protein, plotted by its regression coefficient (x-axis) and VIP score (y-axis). The blue dashed line marks VIP = 1. Red points (VIP > 1) are labeled; gray points (VIP < 1) contribute below-average importance. Proteins in the upper-right quadrant (e.g., CD4, GZMB, ICOS, Ki67) are positively associated with CD8A; proteins in the upper-left quadrant (e.g., pSTAT3, CD68, HLA-DR) are negatively associated. See also Figures S1–S3.

**Figure 2:**
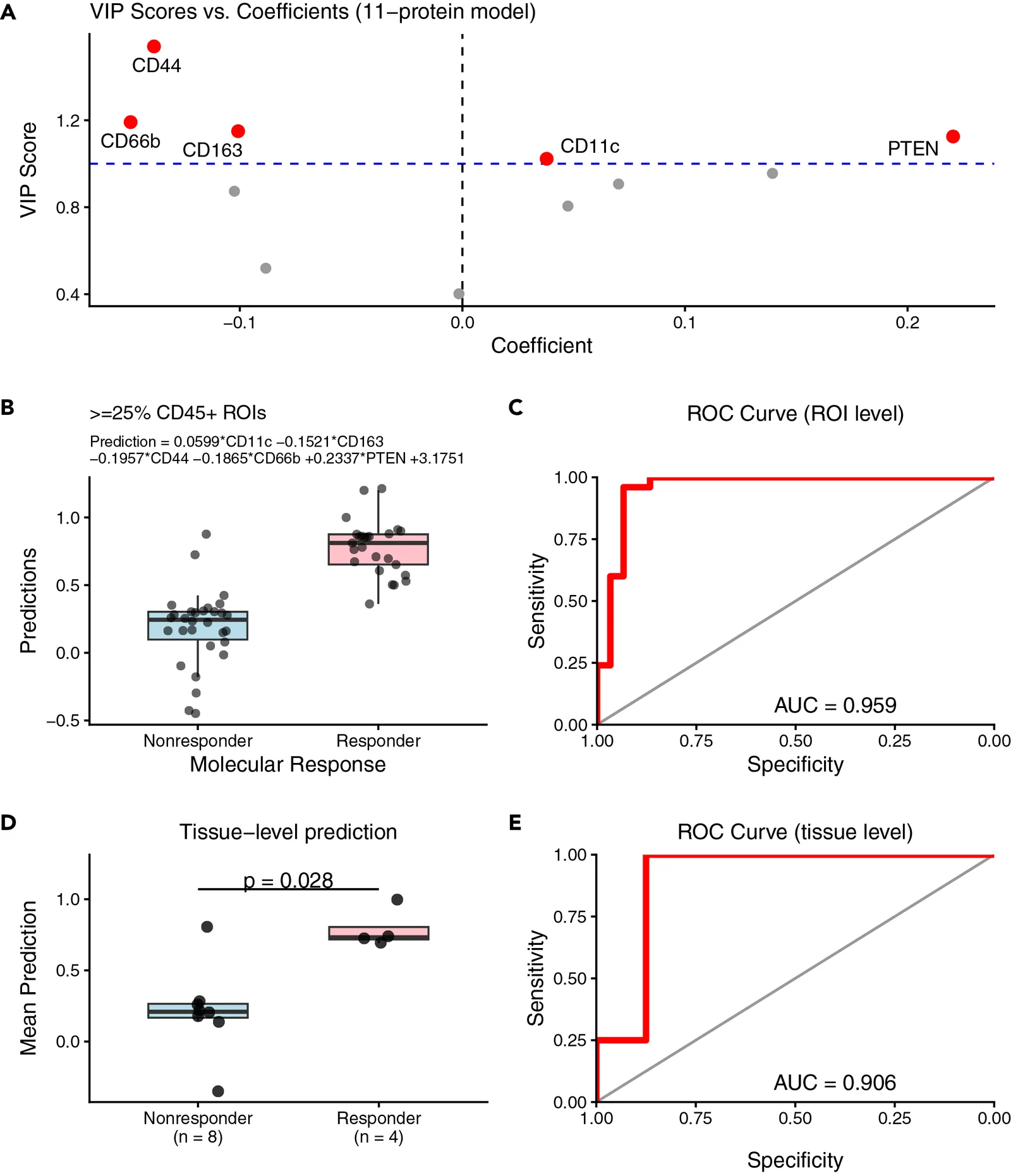
Five-protein signature distinguishes anti-PD-1 responders from nonresponders in immune-rich GBM ROIs with tissue-level validation (A) VIP-coefficient plot for the 11-protein PLS-DA model. Red points above VIP > 1 are the five proteins used in the final prediction model: CD44, CD66b, and CD163 (negative coefficients, associated with nonresponse) and CD11c and PTEN (positive coefficients, associated with response). (B) Distribution of PLS-DA prediction scores for nonresponder and responder ROIs (>=25% CD45+ cells). The linear prediction equation is shown above the panel. The horizontal line in each box represents the median sample value, the ends of each box represent the 25th and 75th percentiles, and the whiskers extend from the ends of each box to 1.5 times the interquartile range. Individual data points are overlaid. (C) ROC curve at the ROI level (AUC = 0.959). (D) Tissue-level validation. Per-ROI prediction scores are averaged within each tissue (sample ID) and compared between nonresponders (n = 8) and responders (n = 4) by two-sided Mann-Whitney U test (p = 0.028). The horizontal line in each box represents the median sample value, the ends of each box represent the 25th and 75th percentiles, and the whiskers extend from the ends of each box to 1.5 times the interquartile range. Individual data points are overlaid. (E) ROC curve at the tissue level (AUC = 0.906).

**Figure 3:**
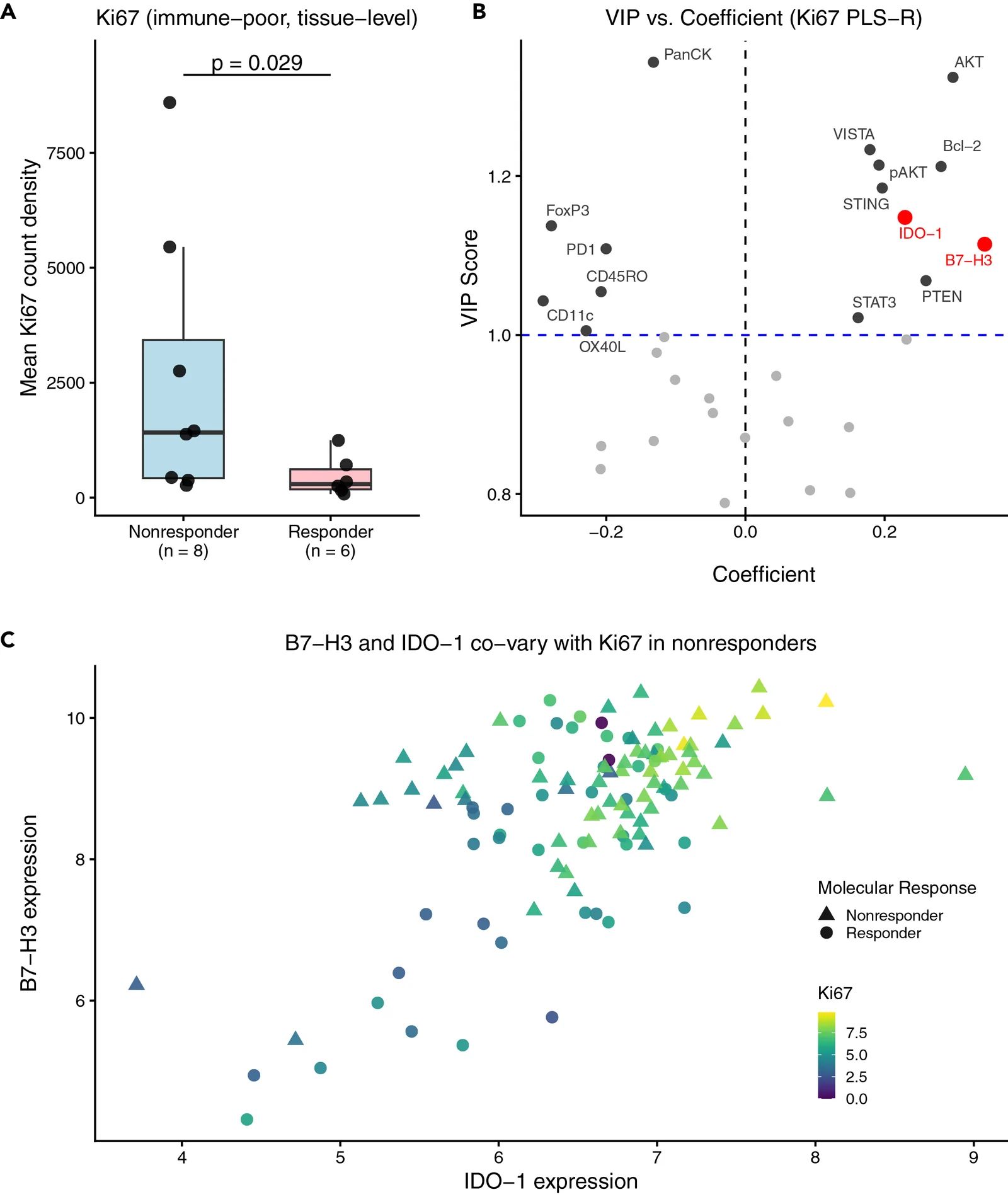
PLS-R reveals potential co-targets for neoadjuvant anti-PD-1 therapy in immune-poor GBM ROIs (A) Tissue-level Ki67 expression (mean count density) in immune-poor ROIs (<25% CD45+ cells), compared between nonresponders (n = 8) and responders (n = 6) by two-sided Mann-Whitney U test (p = 0.029). Ki67 is the only protein in the panel that reaches significance in a univariate comparison. The horizontal line in each box represents the median sample value, the ends of each box represent the 25th and 75th percentiles, and the whiskers extend from the ends of each box to 1.5 times the interquartile range. Individual data points are overlaid. (B) VIP-coefficient plot for PLS-R with Ki67 as the response variable. B7-H3 and IDO-1 (red points) have positive coefficients and VIP > 1, indicating that higher expression of these immune checkpoint proteins is associated with higher Ki67 in nonresponder tissues. (C) Scatter plot of B7-H3 versus IDO-1 expression across all immune-poor ROIs. Color encodes Ki67 expression (viridis scale), and shape distinguishes molecular response groups (triangle = nonresponder, circle = responder). Nonresponder ROIs cluster in the high-B7-H3, high-IDO-1, high-Ki67 region.

**Figure 4:**
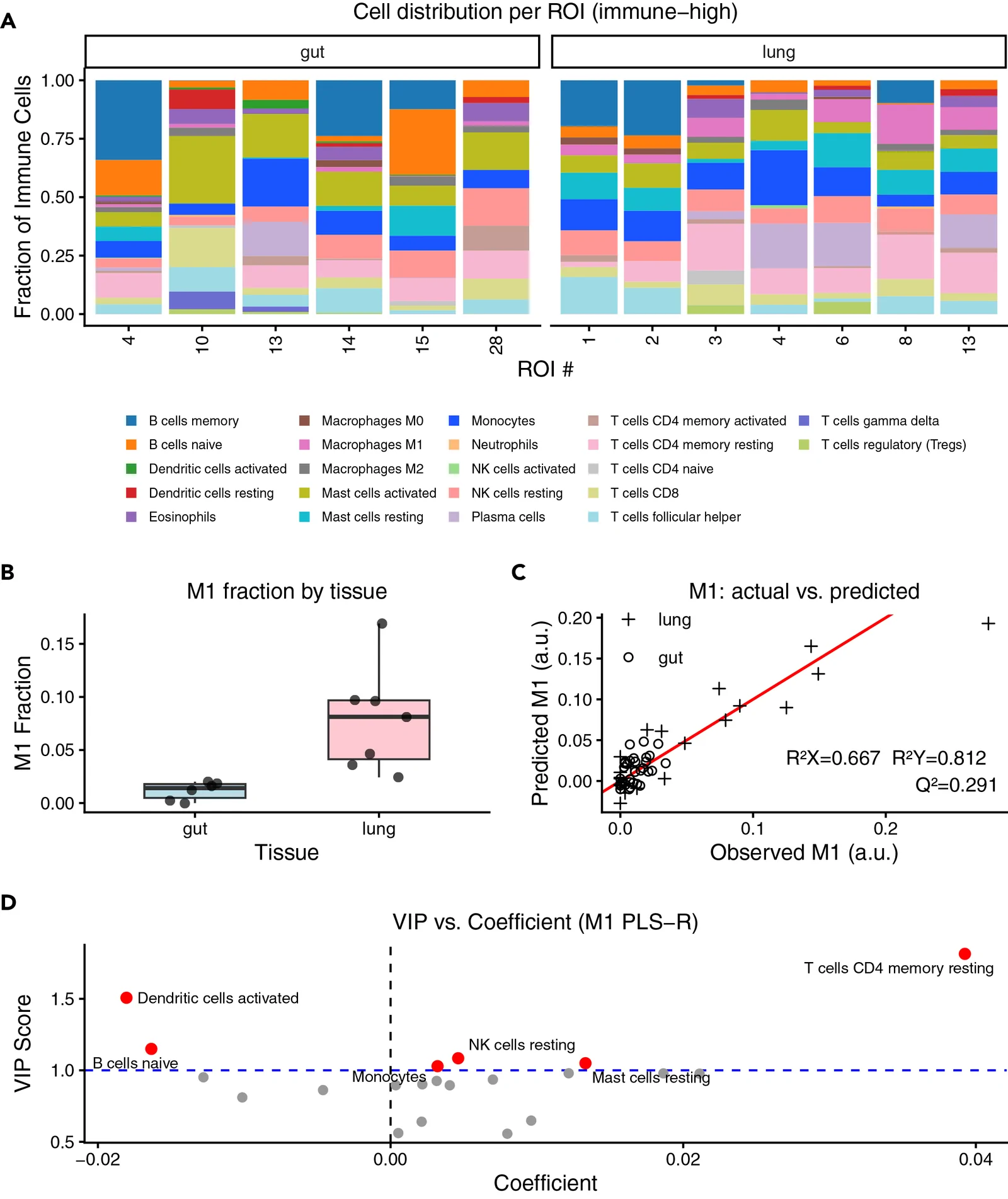
PLS-R identifies cell-type correlates of M1 macrophage abundance in COVID-19 lymphoid tissues (A) Stacked bar chart of CIBERSORTx-derived immune cell fractions for immune-high ROIs, separated by tissue (gut and lung). Each bar represents one ROI and sums to one. (B) Box plot of M1 macrophage fractions by tissue. M1 fractions are consistently higher in lung than in gut lymphoid tissue. The horizontal line in each box represents the median sample value, the ends of each box represent the 25th and 75th percentiles, and the whiskers extend from the ends of each box to 1.5 times the interquartile range. Individual data points are overlaid. (C) Observed versus predicted M1 macrophage abundance (a.u.) across all 53 ROIs (lung and gut combined). Points are shape-coded by tissue (cross = lung, circle = gut). The red line is the identity line. Model-quality metrics are annotated. (D) VIP-coefficient plot. T-cells CD4 memory resting (positive coefficient, upper-right) and Dendritic cells activated (negative coefficient, upper-left) are the top features above VIP > 1 (red points). The negative association between activated dendritic cells and M1 macrophages suggests that inflammatory macrophages may displace dendritic cells from lymphoid tissue in the COVID-19 lung.

### Determining multivariate correlates of CD8 T cells in immune-rich GBM ROIs (worked example 1)

**Timing: 15 min**

This example uses PLS-R to identify which proteins co-vary with CD8A, a cytotoxic T-cell marker, in immune-rich GBM ROIs. The data come from Lu et al.,^1^ who profiled 168 ROIs across GBM tumors from patients treated with neoadjuvant anti-PD-1; ROIs with >=25% CD45+ cells are classified as immune-rich.

***Note:*** The first three worked examples analyze the spatial protein dataset from Lu et al.^1^ (see expected outcomes for details), where ROIs are obtained from tumors classified as either molecular responders (mR) or nonresponders (mNR). This molecular response is defined by a 23-gene transcriptomic signature from Cloughesy et al.^10^

1. Open ‘R/Figure 1.R’ in RStudio. The script reads ‘Nat commun data/Raw data.xlsx’, sheet “GBM” (168 ROIs across 14 GBM tumors from Lu et al.^1^).
2. Run the script by clicking Source in RStudio, or by entering the following in the console.
  source(here::here(“R”, “Figure 1.R”))
  a. The script: filters the 168 ROIs to the immune-rich subset (Ratio of CD45+ cells >= 0.25). To change this threshold for your own data, edit the value on the filter line near the top of the script.
  b. Assembles the predictor matrix X (all measured proteins except CD8A and CD3) and the response vector Y (CD8A).
  c. Fits a PLS-R model with up to 20 components and 10-fold cross-validation, then extracts predictions at 8 components.
  d. Computes VIP scores using the custom pls_vip() function and writes Figure 1.pdf to the ‘figures/’ folder.
    ***Note:*** The core modeling steps are shown below so they can be adapted to your own data. Edit the stratification threshold, the response variable Y, and the predictor set X to match your dataset; the remaining lines are reusable across analyses.
    ## Core PLS-R modeling steps (adapt X, Y, and thresholds to your data)
    gbm_ir <- gbm %>% filter(‘Ratio of CD45+ cells’ >= 0.25) # stratify ROIs
    X <- gbm_ir %>% select(-c(1:5)) %>% select(-CD8A, -CD3) # predictors
    Y <- gbm_ir$CD8A
    # response (outcome)
    plsr.mod <- plsr(Y ∼., data = data.frame(Y = Y, X),
    ncomp = 20, validation = “CV”, scale = TRUE)
    ncomp_use <- 8 # choose from the MSEP plateau
    vip_vals <- pls_vip(plsr.mod, ncomp_use) # VIP scores (custom helper)
    coefs <- coef(plsr.mod, ncomp = ncomp_use) # PLS regression coefficients
3. Open ‘figures/Figure 1.pdf’ to examine the output. The figure contains three panels (A, B, C) described below.
4. Examine Panel A (MSEP curve).

***Note:*** MSEP (mean squared error of prediction) measures how well the model predicts held-out observations at each number of components. The cross-validation (CV) error (dashed line) flattens over a broad range of components, and ncomp_use = 8 lies within it. If the CV curve is still declining at the largest number of components tested, increase ncomp_max and re-run. When applying to your own data, identify where the CV error plateaus, try several ncomp_use values in that range, and choose one from within it. Set a seed before comparing candidates, because the value that maximizes Q^2^ can change between runs when folds are assigned at random. Troubleshooting 3 and troubleshooting 4.

5. Examine Panel B (observed vs. predicted CD8A).

***Note:*** Verify that points fall close to the identity line and that Q^2^ is close to R^2^Y, indicating the model is not overfitting. If the fit is poor, check whether the data were loaded and filtered correctly. Three model-quality metrics are annotated^3^: R^2^X (variance in predictors captured by the components), R^2^Y (variance in CD8A explained), and Q^2^ (variance in CD8A predicted by cross-validation). To explore whether a different ncomp_use improves Q^2^ for your own data, edit the value and re-run. Troubleshooting 4, troubleshooting 5, and troubleshooting 6.

6. Examine Panel C (VIP-coefficient plot).

***Note:*** This is the primary output. Each point is one protein, plotted at its regression coefficient (x-axis) versus its VIP score (y-axis). The blue dashed line at VIP = 1 separates above-average from below-average importance. Proteins in the upper-right quadrant (high VIP, positive coefficient) are positively associated with CD8A. In the demonstration data, these include CD4, GZMB, ICOS, Ki67, CD45RO, CD20, and Bcl-2. Proteins in the upper-left quadrant (high VIP, negative coefficient) are inversely associated with CD8A. In the demonstration data, pSTAT3, CD68, HLA-DR, and STING appear here. The biological interpretation of these associations is discussed in expected outcomes. Troubleshooting 7.

### Classifying anti-PD-1 treatment response in immune-rich GBM ROIs with tissue-level validation (worked example 2)

**Timing: 15 min**

This example uses PLS-DA to identify which proteins distinguish molecular responders from nonresponders in immune-rich GBM ROIs, then validates the model at the tissue level. Predictors were iteratively removed (VIP < 0.8 and |coefficient| < 0.1) across successive models until five proteins remained (see Lu et al.^1^); the protocol demonstrates the final two stages of this reduction.

7. Open ‘R/Figure 2.R′ in RStudio. The script reads two pre-prepared input files from ‘Nat commun data/':
  a. ‘Raw data_plsda round 1.xlsx’ contains the 11 proteins that remained after iterative variable selection from the full 35-protein panel. This is the input to the PLS-DA model shown in Panel A.
  b. ‘Raw data_plsda round 2.xlsx’ contains the per-ROI predictions from the final 5-protein model (Panels B through E).
8. Run the script by entering the following in the console:
  source(here::here(“R”, “Figure 2.R”))
  a. The script fits a 3-component PLS-DA on the 11 remaining proteins, with the binary molecular-response label (pls_code: 0 = nonresponder, 1 = responder) coded numerically as the response variable.
9. Open ‘figures/Figure 2.pdf’ to examine the output (5 panels, A through E).
10. Examine Panel A (VIP-coefficient plot for the 11-protein model).

***Note:*** Five proteins are highlighted in red above the VIP > 1 threshold: CD11c, CD163, CD44, CD66b, and PTEN. These five proteins form the final prediction model used in Panels B through E.

11. Examine Panels B and C (ROI-level performance).

***Note:*** Panel B shows the distribution of prediction scores from the 5-protein model for nonresponder and responder ROIs. The two distributions should be visibly separated, with the prediction formula displayed in the subtitle. Panel C shows the ROI-level receiver operating characteristic (ROC) curve, which plots sensitivity against 1 minus specificity across all classification thresholds; the area under the curve (AUC) summarizes overall discriminatory performance, where 1.0 is perfect and 0.5 is chance. The expected AUC is approximately 0.95. If the AUC is substantially lower on your own data, this may reflect a weaker or absent treatment-response signal in the immune-rich compartment.

12. Examine Panels D and E (tissue-level validation).

**CRITICAL:** This is the critical validation step as multiple ROIs from the same tissue are not statistically independent, so the ROI-level model must be tested at a higher level of aggregation.

***Note:*** The script averages per-ROI predictions within each Sample ID to produce one score per tissue. Panel D shows the tissue-level box plot with the Mann-Whitney p-value annotated (expected p = 0.028, n = 8 nonresponder tissues, n = 4 responder tissues). Panel E shows the tissue-level ROC curve (expected AUC approximately 0.91). The biological interpretation of the five-protein signature and the significance of tissue-level validation are discussed in expected outcomes.

### Revealing potential co-targets for neoadjuvant anti-PD-1 therapy in immune-poor GBM ROIs (worked example 3)

**Timing: 15 min**

This example demonstrates how univariate and multivariate analyses complement each other. Univariate testing identifies Ki67 as the sole protein that differs significantly between responder and nonresponder tissues in immune-poor GBM ROIs; PLS-R then reveals additional proteins that co-vary with Ki67, including checkpoint targets (B7-H3, IDO-1).

13. Open ‘R/Figure 3.R′ in RStudio. The script reads ‘Nat commun data/Raw data.xlsx’, sheet “GBM” (the same data file used in worked example 1).

***Note:*** The following key parameters are described near the top of the script: (1) The immune-poor filter: Ratio of CD45+ cells < 0.25. This is the complement of the immune-rich threshold used in worked example 1. To adjust this threshold for your own data, edit the value on the filter line. (2) ncomp_use = 3. This is the number of PLS components used for the Ki67 model. As in worked example 1, this value was selected from the range where the cross-validation error stops improving. Troubleshooting 3.

14. Run the script by entering the following in the console:
  source(here::here(“R”, “Figure 3.R”))
  a. The script: filters the 168 ROIs to the immune-poor subset and prints the number of ROIs and tissues to the console.
  b. Back-transforms the ln(count + 1)-transformed Ki67 values into linear count density, averages within each Sample ID, and runs a two-sided Mann-Whitney U test comparing molecular responders and nonresponders at the tissue level.
  c. Fits a PLS-R model with Ki67 as the response variable and all other measured proteins as predictors, then computes VIP scores.
  d. Plots B7-H3 against IDO-1 for each immune-poor ROI, with Ki67 expression encoded on a viridis color scale and shape distinguishing molecular response groups, to visualize whether the two checkpoint proteins identified by PLS-R track with proliferation activity.
  e. Writes ‘Figure 3.pdf’ to the ‘figures/' folder.
15. Open ‘figures/Figure 3.pdf’ to examine the output (3 panels, A through C).
16. Examine Panel A (tissue-level Ki67 box plot).

***Note:*** The expected result is p = 0.029 (n = 8 nonresponder tissues, n = 6 responder tissues), consistent with the results in Lu et al.,^1^ Figure 4A. Ki67 is the only protein out of the full panel that reaches significance in a univariate comparison of immune-poor regions. A conventional analysis would stop here.

17. Examine Panel B (VIP-coefficient plot, PLS-R with Ki67 as outcome).

***Note:*** B7-H3 and IDO-1 are highlighted in red above the VIP > 1 threshold with positive coefficients. Neither protein is individually significant in a univariate test, yet both emerge as important in the multivariate model. To explore how the number of components affects the VIP ranking, edit ncomp_use in the script and re-run. Troubleshooting 7.

18. Examine Panel C (B7-H3 vs. IDO-1 scatter plot).

***Note:*** IDO-1 is on the x-axis, B7-H3 on the y-axis, color encodes Ki67 expression (viridis scale), and shape distinguishes molecular response groups. Nonresponder ROIs (triangles) cluster in the high-IDO-1, high-B7-H3 region and carry the highest Ki67 values. The biological and therapeutic implications of this co-variation are discussed in expected outcomes.

### Identifying cell-type correlates of macrophage-driven inflammation in COVID-19 lymphoid tissues (worked example 4)

**Timing: 20 min**

This example extends the workflow to computationally derived cell-type abundances from transcript data. PLS-R identifies which immune cell types co-vary with M1 macrophage abundance across 53 ROIs in COVID-19 lung and gut tissue (Ng et al.^2^).

***Note:*** Cell-type abundances were estimated by CIBERSORTx^7^ (absolute mode, LM22 signature matrix, 1,000 permutations, B-mode batch correction). The pre-computed abundances are included in the repository (‘Cell Rep data/'); CIBERSORTx does not need to be re-run. If applying to your own data, any deconvolution method producing per-ROI cell abundances can be substituted. CIBERSORTx requires free academic registration at https://cibersortx.stanford.edu.

19. Open ‘R/Figure 4.R′ in RStudio. The script reads two files from ‘Cell Rep data/’:
  a. ‘absolute cell fraction.csv’ contains CIBERSORTx-derived immune cell abundances for immune-high ROIs only (used for Panels A and B). This file is a subset of the full 53-ROI file below, filtered to the 13 immune-high ROIs (7 lung, 6 gut). The immune categorization is not performed in this script; it was determined in Ng et al.^2^ based on lymphoid tissue morphology confirmed by *PTPRC* counts and CIBERSORTx immune scores (see Note below).
    ***Note:*** In Ng et al.,^2^ ROIs were selected to include a mixture of lymphoid aggregates and non-lymphoid tissue in each organ. Lymphoid tissue was identified by dense cellular packing in fluorescent staining and confirmed computationally using *PTPRC* transcript counts (encoding CD45) and the CIBERSORTx absolute immune score. ROIs with high values in both measures were categorized as immune-high.
  b. ‘whole cell fraction.xlsx’ contains the same CIBERSORTx output for all 53 ROIs across lung and gut, including both immune-high and immune-low regions (used for the PLS-R model in Panels C and D).
    ***Note:*** The key parameter ncomp_use = 8 near the middle of the script is the number of PLS components for the M1 macrophage model. As in the previous examples, this value can be adjusted by examining the MSEP plateau and comparing Q^2^; raise ncomp_max above it first, since it caps the number of components fitted. Troubleshooting 3.
    **CRITICAL:** If you are applying this example to your own CIBERSORTx output, update the file paths (ABS_FILE and ALL_FILE) at the top of the script. The expected column structure is Tissue, ROI, Mixture, P-value, Correlation, RMSE, Absolute score (sig.score), and one column per cell type.
20. Run the script by entering the following in the console:
  source(here::here(“R”, “Figure 4.R”))
  a. The script: reads the immune-high cell abundances and plots a stacked bar chart (Panel A) and M1 macrophage box plot by tissue (Panel B).
  b. Reads the full 53-ROI cell abundances, fits a PLS-R model with M1 macrophage abundance as the response variable and the other 21 cell types as predictors, and computes R^2^X, R^2^Y, Q^2^, and VIP scores.
  c. Writes ‘Figure 4.pdf’ to the ‘figures/' folder.
21. Open ‘figures/Figure 4.pdf’ to examine the output (4 panels, A through D).
22. Examine Panels A and B.

***Note:*** Panel A shows a stacked bar chart of immune-cell fractions per ROI, separated by tissue (lung and gut). This visualizes the heterogeneity of immune composition across ROIs and organs. Panel B shows a box plot of M1 macrophage fractions by tissue. M1 is consistently higher in lung than in gut, motivating the choice of M1 as the response variable for the multivariate model.

23. Examine Panels C and D (PLS-R model for M1 macrophage abundance).

***Note:*** Panel C plots observed versus predicted M1 abundance, shape-coded by tissue. Points should cluster along the identity line, confirming that the 21-cell-type signature reproduces M1 abundance across both organs. Lung ROIs show higher M1 abundances; gut ROIs cluster near zero. Panel D is the VIP-coefficient plot. Cell types above the VIP > 1 threshold are the populations that jointly co-vary with M1 macrophage abundance. In the demonstration data, T-cells CD4 memory resting (positive coefficient) and Dendritic cells activated (negative coefficient) are among the top features. The biological interpretation of these associations is discussed in expected outcomes. Troubleshooting 5 and troubleshooting 6.

## Expected outcomes

The four worked examples each produce a figure that illustrates one application of the micro-tissue analysis workflow. Running each R script generates the corresponding figure panels and prints summary statistics to the R console. The expected outputs and their biological interpretation are described below.

### Worked example 1: Determining multivariate correlates of CD8 T cells in immune-rich GBM ROIs

This example applies PLS-R to predict CD8A protein expression from the remaining measured proteins (except CD3) in immune-rich ROIs (those with >=25% CD45+ cells). Figure 1A shows the MSEP curve; the cross-validation CV error flattens over a broad range of components, and the selected ncomp_use = 8 lies within it. Figure 1B plots observed versus predicted CD8A values; points should fall close to the identity line. The model-quality metrics annotated on the panel (R^2^X, R^2^Y, Q^2^; see Wold et al.^3^) should show Q^2^ close to R^2^Y, indicating the model is not overfitting.

The primary output is the VIP-coefficient plot (Figure 1C). Proteins above the VIP > 1 threshold with positive coefficients (upper-right quadrant) include CD4, GZMB (granzyme B), ICOS, Ki67, CD45RO, CD20, and Bcl-2. These are canonical markers of T-cell activation, cytotoxicity, and co-stimulation, all expected to co-localize with CD8+ T-cells in an immune-rich tumor microenvironment. Their recovery confirms that the PLS-R model captures biologically coherent relationships. Proteins with negative coefficients above the VIP threshold (upper-left quadrant) include pSTAT3, CD68, HLA-DR, and STING. CD68 is a macrophage marker, and pSTAT3 has been linked to immunosuppression in the literature.^1^ The negative association between these markers and CD8A in the immune-rich GBM microenvironment is consistent with the findings of Lu et al.,^1^ who reported that macrophages and STAT3 signaling may suppress cytotoxic T-cell activity in anti-PD-1-treated GBM. This example therefore serves two purposes: the positive correlates validate the modeling approach against known biology, while the negative correlates reproduce the published mechanistic findings about macrophage-mediated immunosuppression in this tumor context.

### Worked example 2: Classifying anti-PD-1 treatment response in immune-rich GBM ROIs with tissue-level validation

This example applies PLS-DA to classify ROIs from mR versus mNR to neoadjuvant anti-PD-1 therapy. After iterative variable selection reduced the full panel to 11 proteins, a final PLS-DA model identified five with VIP > 1: CD44, CD66b, CD163, CD11c, and PTEN. The VIP-coefficient plot (Figure 2A) shows these five proteins above the importance threshold. In particular, CD44, CD66b, and CD163 have negative coefficients (associated with nonresponse), and PTEN and CD11c have positive coefficients (associated with response). Figure 2B shows the distribution of PLS-DA prediction scores for mR and mNR ROIs; responder ROIs receive higher prediction values. The ROC curve at the ROI level (Figure 2C) yields an AUC of 0.959, confirming strong discriminatory performance.

A critical question for any ROI-level model is whether it generalizes when predictions are aggregated to the tissue or patient level, because multiple ROIs from the same tissue are not statistically independent. Figure 2D addresses this by averaging the per-ROI prediction scores within each tissue (sample ID) and comparing the resulting tissue-level scores between mNR (n = 8) and mR (n = 4). The tissue-level predictions are significantly different between groups (Mann-Whitney p = 0.028), and the tissue-level ROC curve (Figure 2E) yields an AUC of 0.906. This result demonstrates that the five-protein signature identified at the ROI level retains its predictive value when assessed at the clinically relevant tissue level, providing one form of validation.

### Worked example 3: Revealing potential co-targets for neoadjuvant anti-PD-1 therapy in immune-poor GBM ROIs

This example illustrates the central motivation for multivariate modeling. When immune-poor ROIs (those with <25% CD45+ cells) are analyzed by conventional univariate testing, comparing protein expression between mNR and mR tissues, only one protein out of the full panel reaches statistical significance: Ki67, a marker of cell proliferation, is significantly higher in nonresponder tissues (Figure 3A; tissue-level Mann-Whitney p = 0.029, n = 8 mNR and 6 mR). A standard differential expression analysis would stop here, concluding that tumor proliferation is the sole distinguishing feature of treatment resistance in the immune-poor compartment.

PLS-R with Ki67 as the outcome variable reveals a richer picture (Figure 3B). Multiple proteins exceed the VIP > 1 threshold, identifying features that collectively predict Ki67 expression. Among them, B7-H3 and IDO-1 (highlighted in red in Figure 3B) have positive coefficients, indicating that higher expression of these immune checkpoint proteins is associated with higher Ki67 in nonresponder tissues. Figure 3C visualizes this relationship directly: plotting B7-H3 versus IDO-1 across all immune-poor ROIs, with color encoding Ki67 expression, shows that nonresponder ROIs (triangles) cluster in the high-Ki67, high-B7-H3, high-IDO-1 region of the plot.

This finding would not have emerged from testing each protein independently. It generates a testable hypothesis: in tumors that fail to respond to neoadjuvant anti-PD-1 therapy, proliferating tumor cells in the immune-poor compartment co-express alternative immune checkpoint targets, suggesting that combination strategies incorporating anti-B7-H3 or IDO-1 inhibition alongside anti-PD-1 could be explored in future preclinical studies. This example demonstrates how multivariate modeling can move beyond describing what changed to generating hypotheses about why it changed and what to do about it.

### Worked example 4: Identifying cell-type correlates of macrophage-driven inflammation in COVID-19 lymphoid tissues

This example extends the workflow from measured protein expression to computationally derived immune cell-type abundances. CIBERSORTx estimates the abundance, in arbitrary units, of 22 immune cell types in each ROI from the spatial transcript expression matrix. Panels A and B show each ROI’s composition as fractions of its own total immune signal, and the model in panels C and D uses the cell-type abundances before that normalization. Figure 4A displays the immune-cell composition of immune-high ROIs as stacked bar charts, separated by tissue (lung and gut), revealing the heterogeneity of immune composition across ROIs and organs. Figure 4B shows that M1 macrophage fractions are consistently higher in lung lymphoid tissue than in gut, even though both organs harbor SARS-CoV-2. This organ-specific difference motivates the choice of M1 as the response variable for the multivariate model. The gut serves here as a comparator because SARS-CoV-2 also elicits a mucosal immune response in the gut that underlies the gastrointestinal symptoms seen in many patients.

PLS-R is then applied to predict M1 macrophage abundance from the other 21 immune cell types across all 53 ROIs (both tissues combined). Figure 4C plots observed versus predicted M1 values; lung ROIs have higher observed and predicted M1 abundances, while gut ROIs cluster near zero. The VIP-coefficient plot (Figure 4D) identifies the immune cell types most strongly associated with M1 macrophage abundance. T-cells CD4 memory resting appear in the upper-right quadrant (positive coefficient, high VIP). Dendritic cells activated appear in the upper-left quadrant (negative coefficient, high VIP), indicating an inverse relationship: where M1 macrophages are abundant, activated dendritic cells are depleted. Since dendritic cells play a crucial role in activating T-cells in lymphoid tissues, their absence in the lung lymphoid tissues may compromise the local adaptive immune response.^2^ This finding was corroborated in an independent cohort of five COVID-19 lung autopsy tissues from Desai et al.,^11^ where 3 of 5 cases showed enriched inflammatory macrophages and all 5 showed depleted activated dendritic cells in immune-high regions, while none of three non-COVID control tissues exhibited these patterns.^2^ The PLS-R model thus reveals a mechanism by which inflammatory macrophages may displace dendritic cells from lymphoid tissue, disrupting antigen presentation and contributing to the dysfunction of the adaptive immune response in COVID-19 lung.

Together, the four worked examples demonstrate how the micro-tissue analysis workflow identifies biologically meaningful signatures across different biological questions, PLS variants (PLS-R and PLS-DA), data types (protein and transcript), and stratification approaches (image-derived CD45 ratios and computational deconvolution). The biological questions range from identifying correlates of a continuous marker (Figure 1) and classifying treatment response (Figure 2) to generating hypotheses for therapeutic co-targets (Figure 3) and characterizing organ-specific immune composition (Figure 4). Each example also illustrates a different mode of validation: corroboration with known biology (Figure 1), generalization from ROI-level to tissue-level prediction (Figure 2), generation of a testable hypothesis (Figure 3), and replication in an independent cohort (Figure 4).

### Generalization to other spatial platforms

To show that this protocol is applicable to other types of spatial platforms, we applied it to a public 10x Genomics Visium section of human breast cancer.^8^ Visium measures expression at fixed spots, 55 micrometers in diameter and spaced 100 micrometers apart, and thus, the spots were first pooled into micro-tissues in Python by partitioning the section into a square grid and averaging the spots in each square into one pseudo-ROI. Figure S2A shows the grid over the tissue section, with each pseudo-ROI colored by its mean *CD8A* expression, and Figure S2B magnifies one pseudo-ROI and marks the individual Visium spots that were averaged. We then ran the same PLS-R and VIP analysis as worked example 1, with *CD8A* as the response. Figure S2C shows the MSEP curve, where the cross-validation error reaches its minimum at two PLS components. Figure S2D plots observed versus predicted *CD8A* across the pseudo-ROIs, with R^2^X, R^2^Y, and Q^2^ annotated on the panel. The primary output is the VIP-coefficient plot (Figure S2E). The model recovered the cytotoxic T-cell program that co-varies with *CD8A*, including *GZMA*, *GZMK*, *PRF1*, *NKG7*, *CCL5*, and *PTPRC*, while the luminal epithelial markers *KRT18*, *KRT8*, *TACSTD2*, and *ESR1* had negative coefficients.

## Quantification and statistical analysis

All statistical analyses were performed in R (version 4.0 or later) using the packages listed in the key resources table. PLS-R and PLS-DA models were fitted using the pls package with cross-validation; model quality was assessed by R^2^X, R^2^Y, and Q^2^ (Wold et al.^3^), and feature importance was ranked by VIP scores. Group comparisons (Figures 2D and 3A) were evaluated using two-sided Mann-Whitney U tests. Classification performance (Figures 2C and 2E) was assessed by ROC analysis and AUC using the pROC package.

### Why multivariate modeling?

A standard analysis would test each gene or protein independently (for example, comparing expression between responders and nonresponders with a Mann-Whitney test) and rank features by statistical significance. This works when a single dominant marker drives the biology, but spatial profiling data measure tens to hundreds of features per ROI, many of which co-vary. Testing features one at a time cannot capture how they work together, and can miss signatures that are collectively strong but individually modest. In the third worked example, a univariate comparison of immune-poor regions finds only one protein that reaches statistical significance. A conventional analysis would stop there. Multivariate modeling of the same data reveals additional features that collectively predict its expression, including potential therapeutic targets that would not have been identified by testing each protein individually.

### Multivariate modeling with partial least squares

As introduced in before you begin, PLS finds latent components, which are weighted combinations of all measured features that together best explain the outcome variable. This differs from principal component analysis, where the components capture variation in the features alone. PLS handles two practical challenges common in multiplex spatial profiling experiments: the number of measured features can approach or exceed the number of ROIs, and many features are correlated with one another. Because PLS constructs orthogonal components, correlated features do not cause the instability that they would in ordinary regression. PLS models are particularly useful for hypothesis generation because every feature keeps a loading, meaning features are included in the model regardless of their correlation with other features. Other multivariate methods can model the same ROI by feature matrix. Regularized regression such as LASSO returns one member of each correlated group, which is the appropriate choice for a minimal predictive panel. Machine learning approaches such as random forests capture threshold effects and interactions that a linear model cannot, so they are worth trying where the PLS fit is weak, although they lose accuracy where features greatly outnumber ROIs. In our hands, the key features in PLS models are more resilient to low sample sizes compared to regularized regression or machine learning approaches.

### Interpreting PLS models: The VIP-coefficient plot

Plotting VIP scores (y-axis) against regression coefficients (x-axis) produces a two-dimensional summary: the y-axis ranks overall importance, and the x-axis shows the direction and strength of each feature’s association with Y. Features in the upper-right quadrant are both important and positively associated with the outcome; features in the upper-left are important and negatively associated. This VIP-coefficient plot is the primary interpretive tool and appears in all four worked examples.

### Validation and hypothesis generation

Fitting a model is just the beginning. A PLS model generates a hypothesis about which features matter and how they relate to the outcome, but that hypothesis needs to be evaluated. Depending on the question, this can take several forms: corroboration with known biology (do the important features make biological sense?), generalization to a different level of analysis (does an ROI-level model hold when predictions are aggregated to the tissue or patient level?) or to an independent cohort, or generation of a testable hypothesis for future preclinical or clinical studies. Each worked example describes its own validation approach at the point where it is performed, and the expected outcomes section discusses the biological interpretation in detail.

### Testing a model against chance

To test whether a Q^2^ could have arisen by chance, a permutation test can be run. It shuffles the response (Y), refits the model, and records Q^2^ many times (typically 1,000 or more) to create the null distribution of Q^2^. This distribution represents the Q^2^ expected when there is no real relationship between Y and the predictors (X). An observed Q^2^ above nearly all of that distribution indicates the model has captured real signal. The test is most useful when a result is unexpected or the dataset is small. R/Figure S3.R runs it on worked example 1, where the observed Q^2^ of 0.661 exceeds all 1,000 permuted values (permutation p = 0.001, the lowest p-value 1,000 permutations can produce; Figure S3). A Q^2^ of 0.661 means the model predicts two-thirds of the CD8A variation in ROIs it never saw. In contrast, a Q^2^ of zero would mean it predicts no better than always assuming the answer is the average CD8A value.

### Guarding against instability and overfitting

A PLS model can fit well (high R^2^Y) and still predict poorly on ROIs it was not trained on (low Q^2^).^3^ The strategies below reduce that risk.

• Plan the number of ROIs. The number needed depends on how much the outcome varies across ROIs and how closely the measured features follow it. No single ROI count fits every dataset. The demonstration data analyzed in this protocol provide a starting point for experimental planning. Lu et al.^1^ profiled 168 ROIs across 14 GBM tumors from 13 patients, and the 55 ROIs classified as immune-rich were used in worked example 1. To see how far the ROI count can decrease before the model degrades, it was refit on random subsets of those 55. At 15 ROIs, more than one in five subsets returned a negative Q^2^, meaning the model predicted held-out CD8A no better than always assuming the answer is the average CD8A value. At 25 ROIs, more than nine out of ten subsets returned a positive Q^2^, and the four highest-VIP proteins reappeared in 94% of subsets, whereas the four closest to VIP = 1 reappeared in 60%. These numbers serve as a guideline for deciding how many ROIs to collect, but the right number will differ with the data and the question.
• Strategically choose the predictors for the model. The goal is to include as many relevant predictors as possible, as this will improve the precision of the model.^3^ Features that carry little signal can be dropped. For example, the data from Ng et al.^2^ were filtered from a panel of 1,833 transcripts to 1,315 by removing the transcripts that fell below the limit of detection in most ROIs. Similarly, features that barely change across ROIs may also be dropped because a feature that does not vary cannot explain a varying outcome. Be cautious not to overly trim the features, as this may remove biologically important ones that have modest association with the outcome.^12^ Ng et al.^2^ also converted the filtered features by deconvolving them into 22 immune cell-type abundances, providing an alternative way to interpret the model.
• Cross-validate while developing the model. Cross-validation holds out part of the ROIs while the model is trained, then tests how well the model predicts them. 10-fold cross-validation splits the ROIs into ten groups and predicts each group in turn from a model trained on the other nine. Leave-one-out repeats this with a single ROI held out at a time. 10-fold is the default in the pls package and the better choice for most datasets, because leave-one-out is more variable. For very small datasets, leave-one-out may be appropriate as it trains on more of the data. Hold out whole patients instead of individual ROIs when the model is meant to predict new patients, because ROIs from the same patient are not independent.
• Choose the number of PLS components from the MSEP curve (Figure 1A). The curve plots training error and cross-validation error against the number of components. Take a value from the range where the cross-validation error stops improving.

## Limitations

This protocol begins at the point where spatial profiling data have already been generated, segmented, and exported as per-ROI expression matrices. It does not cover upstream steps such as tissue preparation, probe hybridization, image acquisition, or cell segmentation, which are platform-specific and described in separate protocols (e.g., Kim et al.^5^ for GeoMx DSP). The quality of downstream PLS models depends directly on the quality of the input data, including proper normalization and quality control. The worked examples use demonstration datasets with moderate panel sizes (approximately 35 proteins for GBM and approximately 1,800 transcripts for COVID-19), and the behavior of PLS on much larger feature sets from whole-transcriptome spatial platforms (e.g., GeoMx WTA, MERFISH)^13^^,^^14^^,^^15^ has not been evaluated systematically, beyond the Visium demonstration in Figure S2. Users working with high-dimensional panels should consider whether pre-filtering or dimensionality reduction is necessary before applying PLS.

PLS regression and discriminant analysis are linear methods that cannot capture nonlinear relationships, threshold effects, or higher-order interactions between features. The VIP scores and regression coefficients depend on the number of components retained; different choices of ncomp can shift VIP rankings, particularly for features near the VIP = 1 threshold, and can reverse the coefficient sign of features whose association with the outcome is weak. Some subjectivity remains in the component selection process despite the guidance provided by the MSEP curve and Q^2^ metric. The sample sizes in all four worked examples are typical of spatial profiling studies but small by conventional biostatistical standards, and the resulting models should be treated as hypothesis-generating rather than confirmatory. In worked example 4, the CIBERSORTx deconvolution relies on the LM22 reference signature matrix derived from peripheral blood cell populations, and cell-type abundances estimated by deconvolution may not fully reflect the true cellular composition in spatially resolved tissue compartments.

The original analyses in the source publications (Lu et al.^1^ and Ng et al^2^) were performed in JMP Pro using the NIPALS algorithm, whereas this protocol uses the R pls package, which implements kernel PLS and SIMPLS. Model-quality statistics reported by the R scripts may therefore not match those in the original publications, mainly because software differs in how Q^2^ is defined and in the default cross-validation scheme (see troubleshooting, problem 6). The VIP rankings, coefficient directions, and biological conclusions are consistent across implementations.

The COVID-19 worked example (worked example 4) is based on spatial transcript data from a single autopsy donor,^2^ and its cell-type associations are meant to illustrate the protocol. When applying this workflow to draw biological conclusions, use datasets with enough independent patients to support population-level interpretation, and treat single-subject results as hypothesis-generating. In Ng et al.,^2^ the M1-macrophage/activated-dendritic-cell relationship in worked example 4 was corroborated in an independent cohort of five COVID-19 lung autopsies.^11^

## Troubleshooting

### Problem 1

R package installation fails with dependency or version errors. (Related to step 12, before you begin.)

When installing the required R packages, users may encounter errors related to package dependencies, incompatible versions, or compilation failures, particularly on systems with older R installations or missing system libraries. These errors are the most common barrier to running the protocol for the first time.

### Potential solution

• Ensure that R version 4.0 or later is installed. Older versions may lack support for current package versions. Run R.version.string in the console to verify.
• Update all installed packages before installing new ones by running update.packages(ask = FALSE) in the R console.
• If a specific package fails to compile from source, try installing the binary version by setting options(install.packages.compile.from.source = “never”) before running install.packages().
• On Linux systems, some packages require system-level libraries. Consult the error message for the missing library name and install it using your system package manager (e.g., sudo apt-get install libxml2-dev).

### Problem 2

Scripts fail with “file not found” or path errors. (Related to step 14, before you begin.)

The R scripts use here::here() to construct file paths relative to the project root. If the.Rproj file is not open in RStudio, or if data files have been moved or renamed, the scripts will fail with errors such as “cannot open file” or “no such file or directory.”

### Potential solution

• Verify that the.Rproj file is open in RStudio by checking the project name in the upper-right corner of the RStudio window. If it says “Project: (None),” open the.Rproj file from the repository folder.
• Do not use setwd() manually. The.Rproj file sets the working directory automatically; manual overrides can conflict with here::here().
• If you have reorganized the repository folder, ensure that the subfolder structure matches the original download: “R/” for scripts, “Nat commun data/” for the GBM dataset files, “Cell Rep data/” for the COVID-19 dataset files, “visium_demo/” for the Visium demonstration files, and “figures/” for output.

### Problem 3

MSEP cross-validation curve does not plateau. (Related to step 4, worked example 1; step 13, worked example 3; and step 19, worked example 4.)

When examining the MSEP curve (Panel A of Figure 1), the cross-validation error continues to decline at the maximum number of components (ncomp_max) rather than leveling off. This suggests that the current ncomp_max is too low to capture the full complexity of the data, or that the dataset has an unusually high ratio of informative features to observations.

### Potential solution

• Increase ncomp_max in the script (e.g., from 20 to 30) and re-run to see whether the CV error eventually plateaus. If it does, select ncomp_use within the plateau region.
• If the CV error continues to decline even at high ncomp values, the model may be overfitting. Compare Q^2^ and R^2^Y: if R^2^Y continues to increase but Q^2^ does not, the additional components are fitting noise rather than signal. Select an ncomp from within the range where the CV error has stopped improving. Set a seed first, because the value that maximizes Q^2^ can change between runs.
• For datasets with very few ROIs relative to the number of features, consider reducing the feature set by removing uninformative or redundant features before fitting the PLS model.

### Problem 4

Script produces errors when applied to user’s own data. (Related to steps 4 and 5, worked example 1.)

When adapting the scripts to a new dataset, common errors include mismatched column names, unexpected data types (e.g., character strings in expression columns), or missing metadata columns referenced by the script (e.g., “Sample ID,” “Molecular_Response,” “Ratio of CD45+ cells”).

### Potential solution

• Before running the script, open your data file in R and inspect the column names using colnames(). Ensure that the column names in your file match those referenced in the script, or update the script to use your column names.
• Check for non-numeric values in the expression columns using sapply(X, is.numeric). Non-numeric entries (e.g., “N/A″, “below LOD,” or empty strings) will cause the PLS model to fail. Convert or remove these entries before fitting.
• If your dataset does not include a pre-computed stratification variable (such as the ratio of CD45+ cells), you will need to derive one from your imaging data or computational deconvolution before applying the stratification step.
• Ensure that your response variable (Y) and predictor matrix (X) contain no missing values. Use sum(is.na(X)) and sum(is.na(Y)) to check.

### Problem 5

Poor model fit: Q^2^ is much lower than R^2^Y. (Related to step 5, worked example 1, and step 23, worked example 4.)

A large gap between R^2^Y (training fit) and Q^2^ (cross-validated fit) indicates that the model is overfitting the training data and does not generalize well to held-out observations. This can occur when too many components are retained, when the sample size is small, or when the predictors do not contain a strong signal for the chosen response variable.

### Potential solution

• Reduce the number of components (ncomp_use). Overfitting is the most common cause of low Q^2^; fewer components often improve cross-validated performance even though training fit decreases.
• Verify that the response variable (Y) has sufficient variation across the ROIs. If Y is nearly constant, no model will achieve a high Q^2^.
• Check whether the stratification is appropriate. For example, if immune-rich and immune-poor ROIs are mixed together in a single model, the biological heterogeneity between compartments can obscure the signal within each compartment.
• For very small datasets, consider using leave-one-out cross-validation (validation = “LOO” in the plsr() call) instead of 10-fold CV, which can be unstable when group sizes are small.

### Problem 6

Model-quality metrics (R^2^X, R^2^Y, Q^2^) do not match published values. (Related to step 5, worked example 1, and step 23, worked example 4.)

When reproducing the worked examples, the R^2^X, R^2^Y, and Q^2^ values reported in the R console may not match those in the original publications (Lu et al.^1^ and Ng et al^2^;). Additionally, Q^2^ may vary between successive runs of the same script.

### Potential solution

• Differences from the published values are expected, for two reasons. Software differs in how Q^2^ is defined: this protocol reports the cross-validated fit of the chosen model, while JMP Pro and SIMCA also report a cumulative Q^2^, which combines the values across components, and reads higher. A published Q^2^ larger than its own R^2^Y is the cumulative form, because a cross-validated fit cannot exceed a training fit. Check which form of Q^2^ you are looking at before making comparisons. The cross-validation scheme also differs: the original analyses used leave-one-out and this protocol uses 10-fold, the pls package default. Setting validation = “LOO” in the plsr() call matches the original scheme for comparison. Regardless, the VIP rankings and coefficient directions are consistent throughout.
• Run-to-run variation in Q^2^ occurs because 10-fold cross-validation randomly assigns observations to folds. To obtain reproducible Q^2^ values, add set.seed(42) (or any fixed integer) before the plsr() call. This does not affect the model itself, only the fold assignments used for cross-validation.
• The VIP-coefficient plot (the primary interpretive output) is deterministic and does not change between runs; only the cross-validated Q^2^ metric is affected by fold randomization.

### Problem 7

VIP rankings change when ncomp is adjusted. (Related to step 6, worked example 1, and step 17, worked example 3.)

Features near the VIP = 1 threshold may shift above or below the cutoff when ncomp_use is changed. This is expected behavior: VIP scores are computed across all retained components, so adding or removing a component changes the weight each feature receives. Users may be uncertain whether a feature that appears important at one ncomp value is genuinely important.

### Potential solution

• Test several ncomp values within the MSEP plateau range and note which features consistently appear above VIP > 1 with the same coefficient sign. Features that remain above the threshold and keep their direction across multiple ncomp values are robust findings; features that cross the threshold or reverse sign only at specific ncomp values should be interpreted with caution.
• Focus interpretation on features well above VIP = 1 (e.g., VIP > 1.2) and on the direction of their coefficients rather than on the exact VIP value.
• When reporting results, state the ncomp value used and note that VIP rankings were stable across the plateau range, or flag any features that were sensitive to ncomp selection.

## Resource availability

### Lead contact

Further information and requests for resources should be directed to and will be fulfilled by the lead contact, Yue Lu (yue.lu@pharm.utah.edu).

### Technical contact

Technical questions on executing this protocol should be directed to and will be answered by the technical contacts, Alphonsus H.C. Ng (alphonsus.ng@pharm.utah.edu) and Huiqian Hu (huiqian.hu@utah.edu).

### Materials availability

This study did not generate new materials.

### Data and code availability

• This paper analyzes existing, publicly available data. The GBM dataset is from Lu et al.^1^ and the COVID-19 dataset is from Ng et al.^2^ Both datasets are included in the analysis repository. The human breast cancer Visium dataset used in Figure S2 is from 10x Genomics^8^ and is available at the link in the key resources table.
• All original code has been deposited at Zenodo and is publicly available as of the date of publication at https://doi.org/10.5281/zenodo.19967582. The code is also available on GitHub (https://github.com/DigiPharma/Digipharma-Micro-tissue-analysis).
• Any additional information required to reanalyze the data reported in this paper is available from the lead contact upon request.

## Acknowledgments

This work was supported by institutional start-up funds from the 10.13039/100007747University of Utah, including internal supplements provided through the Immunology, Inflammation & Infectious Disease (3i) Initiative and the Center for Metabolic Health.

## Author contributions

A.H.C.N. and Y.L. conceptualized the study and supervised the project. H.H. implemented the code. A.H.C.N. refined the code. All authors wrote the manuscript.

## Declaration of interests

The authors declare no competing interests.

## Declaration of generative AI and AI-assisted technologies in the writing process

During the preparation of this work, the authors used Claude (Anthropic, San Francisco, CA, USA) and ChatGPT (OpenAI, San Francisco, CA, USA) to increase the clarity of the manuscript. After using these tools, the authors reviewed and edited all content as needed and take full responsibility for the content of the publication.
