# Supplementary material for: Protocol for identifying cellular and molecular signatures from spatial profiling data using micro-tissue analysis
Source: STAR Protoc. 2026 Sep 18;7(4):104825. doi: 10.1016/j.xpro.2026.104825 (PMC13602308; doi:10.1016/j.xpro.2026.104825)
Supplement: Supplementary file 1 [file mmc1.pdf]

A

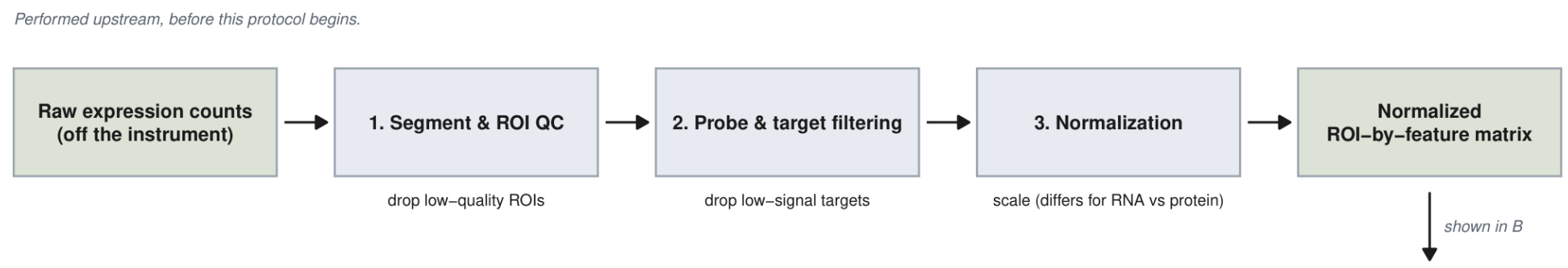

B

ROIs

Metadata					Protein features								
Patient ID	Sample ID	Molecular_ Response	Average No. of Neighbors	Ratio of CD45+ cells	CD8A	CD4	GZMB	ICOS	Ki67	CD45RO	CD20	Bcl–2	•••
G3	ISB004	Nonresponder	2.18	0.265	9.41	9.51	7.23	5.95	6.30	7.57	6.99	6.58	•••
G8	ISB009	Nonresponder	1.66	0.269	5.22	7.29	7.32	4.56	4.61	7.30	3.82	6.24	•••
G4	ISB005	Nonresponder	1.42	0.272	7.21	7.72	7.01	5.46	7.58	7.37	4.83	7.16	•••
G10	ISB014	Responder	1.94	0.272	10.56	9.31	8.40	6.80	7.06	8.62	6.59	7.88	•••
G5	ISB006	Responder	0.64	0.273	8.70	8.80	8.75	5.90	6.90	7.35	1.64	7.83	•••
G3	ISB004	Nonresponder	2.48	0.274	7.45	8.73	6.12	5.10	5.69	6.75	7.43	6.40	•••
G6	ISB007	Nonresponder	1.62	0.300	11.04	10.91	8.43	6.75	6.99	8.60	4.92	7.84	•••
G2	ISB002	Responder	1.15	0.312	9.74	8.97	7.85	5.54	5.97	7.03	5.96	6.37	•••
G6	ISB007	Nonresponder	2.77	0.312	5.56	8.19	8.44	5.70	5.87	9.18	0.00	7.74	•••
G4	ISB005	Nonresponder	0.75	0.320	8.98	8.80	7.39	6.94	7.76	7.13	5.63	8.16	•••
G10	ISB014	Responder	1.67	0.326	10.66	9.03	8.24	6.40	6.91	8.49	6.14	7.32	•••
G8	ISB009	Nonresponder	2.33	0.333	7.07	7.83	7.13	6.09	6.31	7.70	3.22	6.63	•••
⋮	⋮	⋮	⋮	⋮	⋮	⋮	⋮	⋮	⋮	⋮	⋮	⋮	⋮

Figure S1. Upstream quality-control and normalization workflow, and the formatted ROI-by-feature matrix it produces, related to Step 1, worked example 1.

(A) Recommended quality-control and normalization workflow for spatial profiling data. These steps are performed upstream, before this protocol begins, in vendor software (GeoMx DSP Analysis Suite) or with the GeomxTools Bioconductor package; this protocol takes the resulting normalized matrix as its input. Raw expression counts pass through segment and ROI quality control (dropping ROIs with too few nuclei, too little tissue area, or, for a sequencing readout, low sequencing saturation), probe and target filtering (removing outlier probes and dropping targets detected in too few ROIs, using the limit of quantification derived from negative-control probes), and normalization, which differs by modality: third-quartile (Q3) scaling for RNA, and scaling to housekeeping or IgG background controls or to segment area or nuclei count for protein. In the two demonstration datasets, the RNA data (Ng et al.,[S1] COVID-19 transcript) were Q3 scaled with genes below the limit of detection in more than 85% of ROIs removed, and the protein data (Lu et al.,[S2] GBM) were normalized with ERCC spike-in controls, background-subtracted against IgG isotype controls, and scaled to UV-mask tissue area to yield count density.

(B) The formatted ROI-by-feature matrix that results, and that this protocol uses in worked example 1. Rows are ROIs and columns are features. The first five columns are metadata (Patient ID, Sample ID, Molecular_Response, Average No. of Neighbors, Ratio of CD45+ cells) and the remainder are normalized protein expression values from the 35-plex immuno-oncology panel, including CD8A, the response variable in worked example 1. Data are from the Lu et al.[S2] GBM dataset; 12 of 168 ROIs and 8 of 35 panel proteins are shown, the ROIs being the first 12 that pass the immune-rich threshold (Ratio of CD45+ cells at or above 0.25) used in worked example 1. Dots mark the columns and rows not displayed.

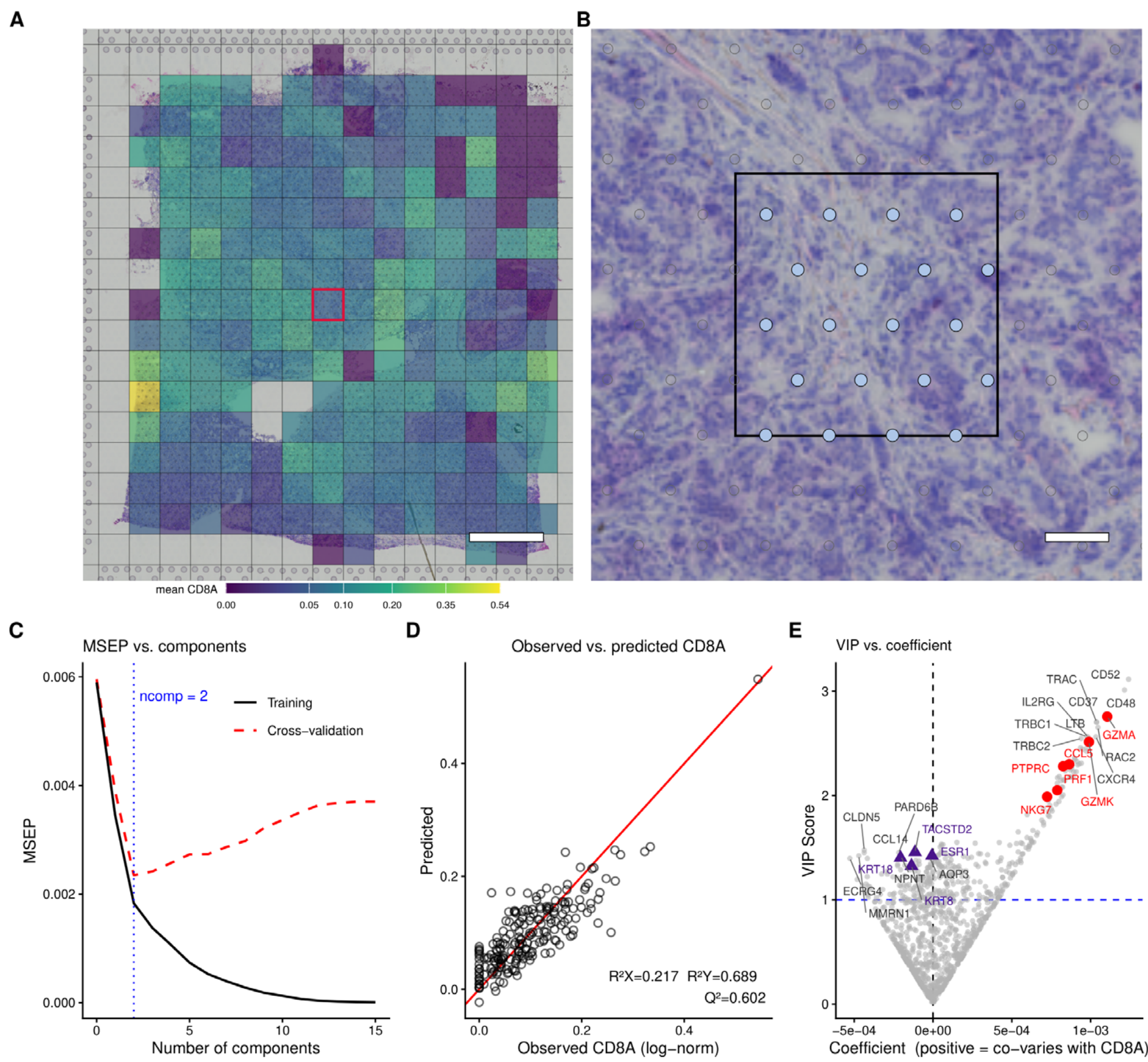

Figure S2. The micro-tissue analysis workflow applied to 10x Genomics Visium data, related to Steps 1 to 6, worked example 1.

Public 10x Genomics Visium section of human breast cancer (10x Genomics, Human Breast Cancer Block A Section 1, Space Ranger 1.1.0, CC BY 4.0).[S3] Spot counts were log-normalized and pooled on a 414 micrometer square grid into 205 pseudo-ROIs, each holding a median of 20 spots (range 8 to 23). Bins containing fewer than eight spots were excluded. PLS-R and VIP analysis follow worked example 1, with *CD8A* as the response Y and all other genes as predictors X, after removing the direct co-markers *CD8B*, *CD3D*, and *CD3E*. Produced by R/Figure S2.R.

(A) Grid over the tissue section, with each pseudo-ROI filled by its mean *CD8A* on a square-root color scale. Excluded bins are unfilled. The red outline marks the pseudo-ROI shown in (B). Scale bar, 1 mm.

(B) The outlined pseudo-ROI at higher magnification. Circles mark the Visium spots that were averaged into it. Scale bar, 100 micrometers, equal to one spot pitch.

(C) Mean squared error of prediction (MSEP) against the number of PLS components. The cross-validation error reaches its minimum at two components.

(D) Observed versus predicted *CD8A* across the pseudo-ROIs ($R^2X = 0.22$, $R^2Y = 0.69$, $Q^2 = 0.60$).

(E) VIP score against regression coefficient for each gene. The blue dashed line marks VIP = 1. Red circles mark the cytotoxic T-cell and immune-infiltration markers, *GZMA*, *GZMK*, *PRF1*, *NKG7*, *CCL5*, and *PTPRC*, which have positive coefficients. Purple triangles mark the luminal epithelial markers *KRT18*, *KRT8*, *TACSTD2*, and *ESR1*, which have negative coefficients.

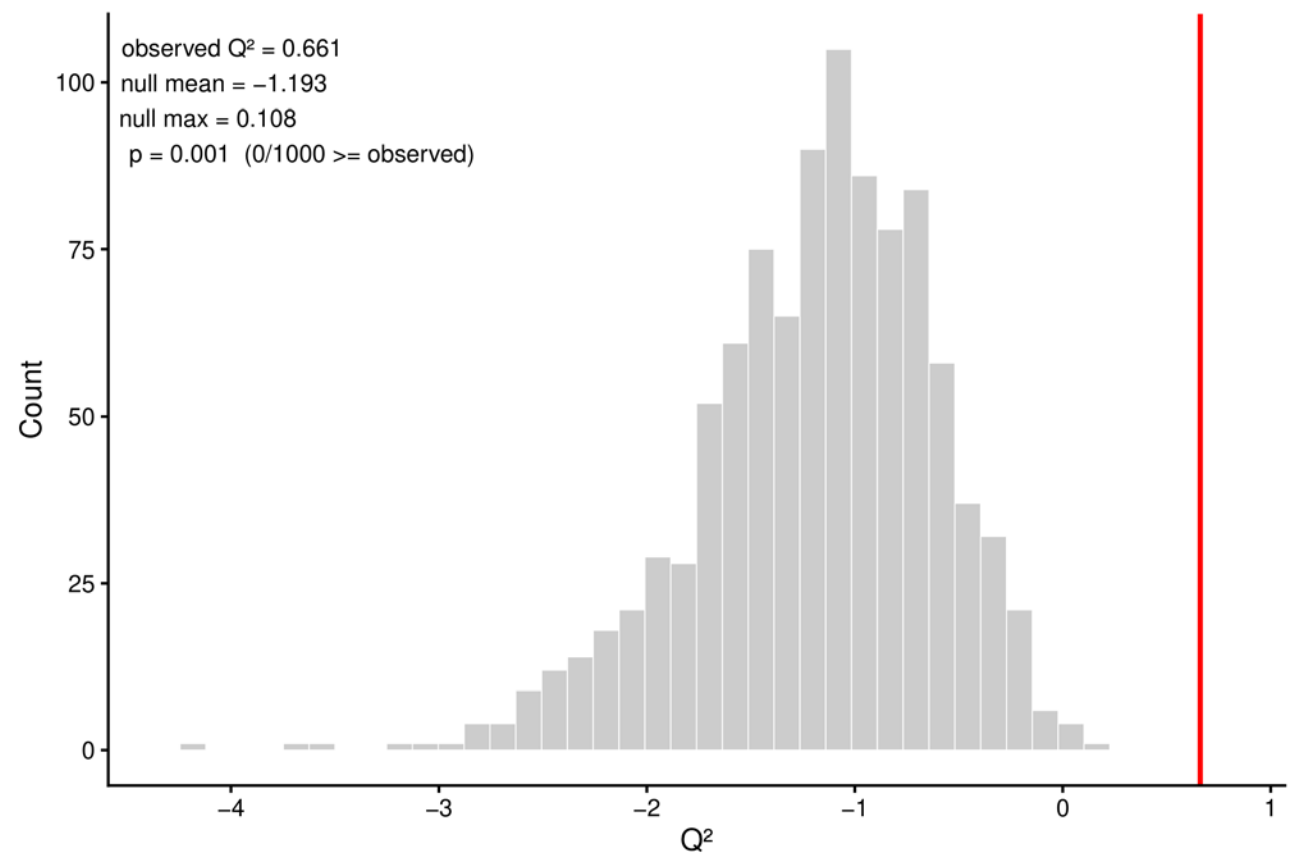

Figure S3. Permutation null distribution of the cross-validated Q^2, related to Step 5, worked example 1.

Q^2 from 1,000 refits of the worked example 1 PLS-R model,[S2] each on a random permutation of the CD8A response (gray bars). The red line marks the observed Q^2 of 0.661 from the unpermuted model. The permuted models give a null mean Q^2 of -1.193 and a null maximum of 0.108. No permutation reaches the observed value, so the permutation p value is 0.001, the lowest that 1,000 permutations can produce. Produced by R/Figure S3.R.

References

S1. Ng, A.H.C., Hu, H., Wang, K., Scherler, K., Warren, S.E., Zollinger, D.R., McKay-Fleisch, J., Sorg, K., Beechem, J.M., Ragaglia, E., et al. (2023). Organ-specific immunity: A tissue analysis framework for investigating local immune responses to SARS-CoV-2. Cell Rep 42, 113212. 10.1016/j.celrep.2023.113212.

S2. Lu, Y., Ng, A.H.C., Chow, F.E., Everson, R.G., Helmink, B.A., Tetzlaff, M.T., Thakur, R., Wargo, J.A., Cloughesy, T.F., Prins, R.M., and Heath, J.R. (2021). Resolution of tissue signatures of therapy response in patients with recurrent GBM treated with neoadjuvant anti-PD1. Nat Commun 12, 4031. 10.1038/s41467-021-24293-4.

S3. 10x Genomics. (2020). Human Breast Cancer (Block A Section 1), Visium Spatial Gene Expression dataset, Space Ranger 1.1.0.
